# Changes in Anxiety following Taste Education Intervention: Fussy Eating Children with and without Neurodevelopmental Disorders

**DOI:** 10.3390/nu15224783

**Published:** 2023-11-15

**Authors:** Sigrun Thorsteinsdottir, Anna S. Olafsdottir, Olof U. Traustadottir, Urdur Njardvik

**Affiliations:** 1 Faculty of Health Promotion, Sport and Leisure Studies, School of Education, University of Iceland, Stakkahlid, 105 Reykjavik, Iceland; annaso@hi.is; 2 Faculty of Psychology, School of Health Sciences, University of Iceland, Saemundargata 12, 102 Reykjavik, Iceland; olofu@landspitali.is (O.U.T.); urdurn@hi.is (U.N.)

**Keywords:** fussy eating, anxiety, neurodevelopmental disorders, ADHD, autism spectrum disorder, behavior change, teaching kitchens, nutrition education, health and wellness, food skills

## Abstract

Despite the surge in studies on fussy eating in recent years, anxiety as an associated factor is generally not considered, even though children with fussy eating and those with neurodevelopmental disorders, including Autism Spectrum Disorder or Attention Deficit/Hyperactivity Disorder (ADHD) often have higher levels of anxiety than typically developing children. The current study investigated changes in anxiety scores during a Taste Education intervention, a seven-week school-based intervention for 71 children with fussy eating. Comparisons were made based on neurodevelopmental status (between children with (*n* = 30) and without (*n* = 41) neurodevelopmental disorders). Participants were paired based on age, sex, and neurodevelopmental disorder. The Multidimensional Anxiety Scale for Children (MASC) was administered at delayed intervention (for those waiting 7 weeks before starting the intervention), pre-intervention, post-intervention, and at six-month follow-up. Results did not indicate elevated anxiety based on mean MASC T-scores. MASC Total T-scores ranged from slightly elevated to average, decreasing significantly between pre-intervention and post-intervention, plateauing at six-month follow-up. Significant reductions between measurement points were seen for the *physical symptoms*, *social anxiety*, and *separation anxiety subscales*, but not for harm avoidance. Repeated measures analysis of variance with neurodevelopmental disorders as between-subjects factors did not reveal a significant interaction effect between neurodevelopmental disorders and changes in MASC Total score or subscales. The results indicated that our food-based intervention did not elevate MASC scores in fussy eating children, with or without neurodevelopmental disorders.

## 1. Introduction

Fussy eating is commonly defined as a child’s reluctance to try new or unfamiliar food, resulting in a diet particularly low in variety [1,2]. Similarly, food neophobia is a child’s avoidance of or refusal to eat unknown foods [1,3]. Food neophobia and fussy eating are considered closely related, and since food neophobia is regarded as a part of fussy eating, both terms will hereafter be referred to as fussy eating. According to longitudinal studies, the prevalence of fussy eating sits between 13% and 22% at any age ranging from three to 11 years old [4,5]. Although challenging eating behaviors are often considered a part of children’s typical development, parents may set unreasonable and harsh rules during dinnertime while their child goes through a fussy eating phase. The reciprocal relationship between the parents’ tension and children’s maladaptive mealtime behavior may cause increased difficulty, stress and even anxiety [6,7,8,9,10,11].

Fussy eating has increasingly been associated with a rise in symptoms of psychopathology, such as anxiety [12,13], as well as neurodevelopmental disorders (ND), including Autism Spectrum Disorder (ASD) or Attention Deficit/Hyperactivity Disorder (ADHD) [14,15,16]. Among other criteria, ASD is defined by obsessive eating patterns, failure to eat the usual family diet, food refusal, inability to accept new foods, and hyper- or hypo-reactivity to sensory experience, which can be represented by a limited diet or fussy eating [17,18]. Rejecting fruit and vegetables is especially common in children with ASD [12,15,19,20,21]. Fussy eating seems to be similar across different NDs, such as ASD and ADHD, even when accounting for comorbidity [19,22,23]. Greater sensitivity to taste and smell often identified in these disorders could explain why these sensitivities are more likely in children with ND than in TD children [19]. Fussy eating is quite common in children with ADHD (17–40%) compared with children without ND [11,22,23,24], where challenging eating behaviors may be represented by impulsivity and overeating sweet and energy-dense foods, regardless of hunger levels, as well as refusing healthier options such as fruit and vegetables [25,26,27]. The onset of ADHD is in childhood and is defined by a persistent pattern of inattention and/or hyperactivity-impulsivity and may interfere with functioning, development, and eating behaviors [28,29,30,31]. Interestingly, 25–50% of 8–12-year-old children with high-functioning ASD have comorbid ADHD [22,32], which may account for some shared symptoms between children with ASD and ADHD. Further, children with ND also commonly share comorbidities, such as anxiety disorders [12,33,34,35], with research showing that children with moderate and severe fussy eating later show increased signs of generalized anxiety disorder, social anxiety, and depression [16]. In one study, moderate and severe fussy eating was associated with significantly elevated symptoms of depression, social anxiety, and generalized anxiety in TD children [16]. In a recent study published by the authors on children with obesity (mean age 12.0 years), the odds of being a fussy eater were increased by a factor of 4.11 when a child had anxiety, after adjusting for medication use. The odds were not raised for children with ND or depression [36]. 

Children with ND have a higher percentage of comorbid anxiety disorders than the general population [37,38,39,40]. For example, children with ASD score significantly higher than a normative sample for various types of anxiety, including physical symptoms, social anxiety, and separation anxiety [41]. Some studies report 25–42% of children with ADHD also have a comorbid anxiety disorder [37,42], with a further study reporting comorbid anxiety disorders in ASD and ADHD over 45% and 19%, respectively [40]. Interestingly, Pliner et al. [43] showed that increased anxiety unrelated to food could increase fussy eating. 

A considerable proportion of the variance in fussy eating can be explained by environmental factors, which may be adjusted with suitable interventions [6,44,45]. However, the authors of this current study identified only one food-based intervention on the role of anxiety in fussy eating, in which greater sensitivity to sensory information explained the relationship between anxiety and selective eating in children [46]. However, children with ND were not considered [22,24,47]. Some of the more successful interventions for fussy eating utilize repeated exposure to disliked or unfamiliar food [44,45,48,49] and results from a recent food-based intervention, Taste Education, showed positive outcomes such as increased food variety, reduced food fussiness for children based on ND status (i.e., with or without ND), and signs of increased enjoyment of eating [44]. Furthermore, reductions in problematic mealtime behaviors were reported, regardless of ND status [45]. The core elements of the Taste Education method are simple and mostly center on repeated exposure to disliked stimuli (i.e., fruit, vegetables, nuts, and seeds), parental education, and a gentle sensory-based approach with no pressure on tasting the foods—a novelty in food-based interventions. Intensive, large-scale interventions for fussy eating have been implemented in different countries with positive results [50,51,52], as well as interventions with less favorable outcomes [53,54,55]. These interventions did not specifically measure anxiety and did not compare children with and without ND. Despite children with fussy eating often experiencing anxiety when confronted with disliked foods [15,16,46], and despite the higher prevalence rates of anxiety in children with ND than TD children [12,21,23,37,38,40,42,56], there is a general lack of research on anxiety and fussy eating in food-based interventions. 

This current study aimed to investigate changes in anxiety scores in children with and without ND during and following their time in the Taste Education program. The first aim was to measure changes in anxiety before and after the Taste Education intervention. It was hypothesized that anxiety scores would decrease after the intervention. The second aim was to compare changes in anxiety scores based on ND status. It was hypothesized that anxiety scores for children with ND would reduce less than for children without ND.

## 2. Materials and Methods

The data used in this study were based on a longitudinal, randomized controlled study of a Taste Education intervention which took place at a home-economics teaching kitchen within the School of Education at the University of Iceland, from 2018 to 2019. Children who completed the MASC (Multidimensional Anxiety Scale for Children) at all timepoints were used in the study. For more detailed information on the original study’s methods, see Thorsteinsdottir et al. [44].

### 2.1. Study Design

Participants were paired based on age, sex, and ND and split randomly into two groups: immediate intervention (*n* = 29) and delayed intervention (*n* = 42). The delayed intervention groups were offered the same program as the immediate intervention groups, only delayed by seven weeks. By the end of the study, all participants had received the Taste Education intervention in order to obtain a comparison group. To verify the diagnoses of ADHD and ASD in children, it was necessary for all children to have received a diagnosis from one of the three primary diagnostic centers in Iceland. Children with anxiety were either diagnosed at the diagnostic centers, or by other reputable, licensed psychologists. These centers employ standardized diagnostic instruments and protocols to ensure consistency and accuracy. Parents were asked about children’s medication status, i.e., whether the children were taking medication, and which type. No significant differences were found in the background measures between the immediate and delayed intervention groups, so the data were pooled in both groups to increase statistical power [44]. For the present study, a chi-square test for association was performed between ND status and all background measures. The only significant associations were between ND status and children’s gender *χ*^2^(1) = 4.280, *p* = 0.039. 

The sessions conducted were non-invasive and posed no risks to the participants. The study utilized good-quality ingredients donated by a local grocery store. There was no obligation for the children or parents to taste any of the food provided, and the participants were free to leave at any time. Ethical approval was obtained from the National Bioethics Committee and the Data Protection Authority (VSNb2017110020/03.01), and the study was performed in accordance with the Declaration of Helsinki.

### 2.2. Participants

Participants were invited to participate through communications across social media, through e-mail lists in partnership with the Icelandic ASD and ADHD societies, and via adverts on a website dedicated to the study. The inclusion criterion comprised fussy eating children and exclusion criteria included children not able to feed themselves and those not able to speak Icelandic. The study was open for children aged 8–12 years old, with and without ND, and their parents. One hundred and ninety parent–child dyads answered the screening questionnaire online about their children and themselves, and those willing to participate also provided informed consent. The intervention did not interfere with other services the children were receiving, nor did it exclude them from receiving them. The study provided contact information for a pediatrician and a psychologist if the parents had any inquiries about any parts of the intervention regarding their child’s mental or general health. The children participating in the Taste Education study were accompanied by their parents or primary caregivers. Although both parents were invited, mothers primarily participated along with their children; fathers, on average, participated in 30% of the sessions. All children attended mainstream schooling. For further details, see Thorsteinsdottir et al. [44]. 

### 2.3. Intervention

Two parent education sessions, two hours each, were scheduled before six kitchen sessions with the parents–child dyads [44]. Approximately 10 dyads were in each group, working on various tasks such as playing food-based games, partaking in sensory experiences, easy food preparation, and cooking/baking. An integral part of the Taste Education was teaching parents to approach their children gently and with awareness regarding their sensory challenges. Parents were taught basic behavior modification techniques (e.g., refraining from negatively reinforcing children’s behavior when apprehensive towards tasks or when responding in opposition to disliked foods). 

The parent–child dyads were introduced to a gentle repeated exposure method to food-based stimuli, using the same ingredients in various shapes and forms and learning about their qualities. Although the methods mainly centered on food, a multisensory approach incorporating sound, touch, sight, smell, and taste was also integrated into the sessions for the sensory experiences. The children were “nudged” gently to approach the stimuli (i.e., various food items) by the parents and taste educators without using pressure. For instance, the parents were instructed to follow examples from the taste educators and say things like, “This sure looks like an interesting texture, wonder what it would sound like when biting into it”, and “Do you think mangoes are sweeter than apples?”, “Do you want to guess and then try?”, “I am going to make a plate with as many colors of the rainbow as possible, do you want to try it too?” Parents, of which some acknowledged being sensitive to certain stimuli [57], were invited to taste food items when offered by a taste educator and positively or neutrally describe the sensory aspects. Two lead taste educators (a psychologist and a nutritionist) and four assistant taste educators assisted in each session. Completion of the Taste Education was rewarded with a certificate and a totem bag, and no other incentives were provided for participating. A more detailed description of the sessions as well as the foods used in the study may be found in our published papers [44,45]. 

### 2.4. Measures

#### 2.4.1. Multidimensional Anxiety Scale for Children (MASC)

**Figure 1:**
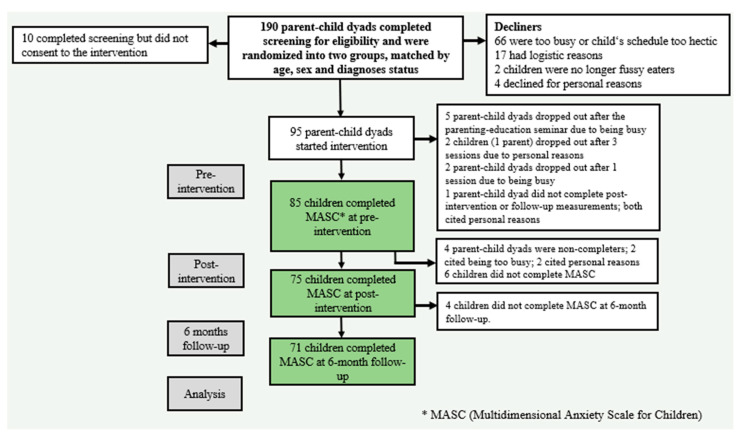
Intervention flow diagram showing stages of the study and measurement points for MASC (Multidimensional Anxiety Scale for Children).

The Icelandic version of the MASC [58] was used to measure participants’ anxiety levels. The scale consists of 39 questions on four subscales: *physical symptoms*, *social anxiety*, *harm avoidance*, and *separation anxiety*. The questions are answered on a four-point Likert scale ranging from *never true* (0) to *very true* (3). The total score can range from 0 to 117. Means were displayed as T-scores with guidelines indicating <40 = low, 40–54 = average, 55–59 = high average, 60–64 = slightly elevated, 65–69 = elevated, and ≥70 very elevated [58]. The MASC questionnaire was translated and standardized in Icelandic in 2004 [59] and has been used for decades to screen and measure anxiety symptoms [60]. The psychometric properties of the Icelandic version are good. The Cronbach’s alpha for internal consistency ranges from 0.86 to 0.91 for the MASC Total score, with lower internal consistency for the subscales. *Physical symptoms and social anxiety* range from 0.74 to 0.84 and are considered acceptable and good, respectively. However, the internal consistency for *harm avoidance* and *separation anxiety* is lower, ranging from 0.62 to 0.74, respectively [59]. For the current sample, the Cronbach alpha for internal consistency was good (0.90) for the MASC Total score. Internal consistency was lower but acceptable and good for the subscales: *physical symptoms*, *social anxiety*, and *separation anxiety*, ranging from 0.73 to 0.85. The Cronbach alpha for internal consistency of the subscale *harm avoidance* was considerably lower, or 0.54, which is considered poor internal consistency. Children answered questions regarding the MASC at the start of the intervention (children in the delayed intervention also answered seven weeks prior) and at post-intervention, with follow-up at six months (Figure 1). Questionnaires were administered and stored online using Qualtrics software (Qualtrics, Provo, UT, USA). 

#### 2.4.2. Background Information

Parents answered questions online regarding various background information (Table 1), i.e., the child’s date of birth, sex, whether the child had ND, the sex of the parent answering the questionnaire, parent education level, employment status, relationship status, how many children lived in the household, and whether they lived in a single-parent home. There were no significant differences in the characteristics between the 81 parent–child dyads who completed the intervention and the 71 children who completed the intervention and all measurement points for the MASC.

### 2.5. Statistical Analysis

Data were analyzed using R Studio 4.2.2 [61]. Descriptive statistics (mean, standard deviation, and percentages) were used to illustrate the characteristics of the study population. Repeated measures analysis of variance (ANOVA) was conducted to measure the changes in anxiety levels on MASC. The 71 participants who completed all measurement points for the MASC (pre- and post-intervention and six-month follow-up) were included in the results; others were excluded listwise. No outliers were detected. Little’s [62] test (MCAR) was not significant, *χ*^2^(272, *N* = 71), *p* = 0.729, so missing values were missing completely at random. Data were normally distributed. As is common, the missing values were replaced using the mean, although this method may degrade the statistical performance of the data [63]. Bonferroni correction was used for post hoc analyses. Main effect for changes in MASC between children with and without ND was assessed using repeated measures ANOVA with ND status as a between-subjects factor and time (measurement points) as a within-subjects factor, and for testing whether there was a significant interaction between ND status and time. When a significant interaction between condition and time was detected, an independent *t*-test was conducted to ascertain whether there were significant differences in the change scores between the two conditions. If no significant interaction between condition and time was observed but a significant time effect was evident, we proceeded with an independent *t*-test between the treatment conditions post-intervention. This was carried out to determine whether the effect of time was attributable to the treatment, both conditions, or a combination of factors. 

## 3. Results

### 3.1. Descriptive Statistics

Out of 190 potential participants, 95 agreed to participate, and 81 parent–child dyads completed the intervention (Figure 1). Those who dropped out stated various reasons for not continuing, e.g., being too busy or living too remotely. For the six-month follow-up, the response rate for the intervention was 93% for the 81 parent–child dyads [44]. Seventy-one children answered the Multidimensional Anxiety Scale for Children (MASC) [58] for all measurement points (88% response rate).

Characteristics of the 71 participants, based on ND status, can be viewed in Table 1. The majority of participants were mothers (91.5%), with the majority (78.9%) having completed a university-level education and 76.1% in a full-time occupation. The mean age of children was 9.2 (*SD* = 1.5) at pre-intervention, and 39.4% were female. Thirty-one children (43.7%) had ND, i.e., ASD, ADHD, or both; 11.3% had anxiety; and 9.8% had other disorders, such as mild learning difficulties or developmental delays. Two children without ND had anxiety, with the remaining six participants split between those with ADHD, or ASD primarily, and those with both NDs.

### 3.2. Changes in Anxiety Scores

A paired sample *t*-test was used to test whether anxiety measurements in the delayed intervention group had changed significantly in the seven weeks from the baseline questionnaire to pre-intervention (while waiting for the intervention to commence). The difference between the two measurement points was not significant, *t*(28) = 0.342, *p* = 0.735. There were also no significant differences in the MASC Total score between the groups for any of the measurement points, i.e., pre-intervention, *t*(28) = −0.334, *p* = 0.740, post-intervention *t*(28) = −0.100, *p* = 0.920, or six-month follow-up *t*(28) = −0.831, *p* = 0.409. The data from the immediate intervention and delayed intervention groups were pooled to estimate differences in treatment based on ND status. Repeated measures ANOVA was used to measure whether changes in MASC scores differed significantly between measurement points, i.e., pre-intervention, post-intervention, and at the six-month follow-up. Changes in MASC are shown in Table 2.

The MASC Total score decreased post-intervention, plateauing at six-month follow-up. The MASC Total T-scores ranged from high average (56.6) at pre-intervention to average (53.1) at six-month follow-up. Scores for the MASC subscales ranged from slightly elevated (62.3) at pre-intervention for *separation anxiety*, to average (47.9) for *physical symptoms* at six-month follow-up. None of the mean MASC T-scores showed elevated anxiety (65–69). The changes in the MASC Total score were significant between at least two measurement points (Table 2). When using the Bonferroni correction, the differences in MASC Total score were significant between the pre- and post-intervention measurements (*p* = 0.047) but not between other measurement points.

Significant differences were found between at least two measurement points on the subscale *physical symptoms* (Table 2). The post hoc test showed significant differences in the *physical symptoms* subscale at pre-, and post-intervention, *p* = 0.049, and between pre-intervention and six-month follow-up, *p* = 0.039. On the *harm avoidance* subscale, the changes were not significant. On the subscale *social anxiety*, significant differences were found between at least two measurement points; however, when using the Bonferroni correction, the differences were not significant. On the subscale *separation anxiety*, there was a significant difference between at least two measurement points. The post hoc test showed significant differences between the *separation anxiety* measure at pre-intervention and six-month follow-up, *p* = 0.026. Effect sizes calculated between pre-intervention and six-month follow-up were small in all instances.

### 3.3. Changes in Anxiety Scores Based on Neurodevelopmental Disorder Status

**Figure 2:**
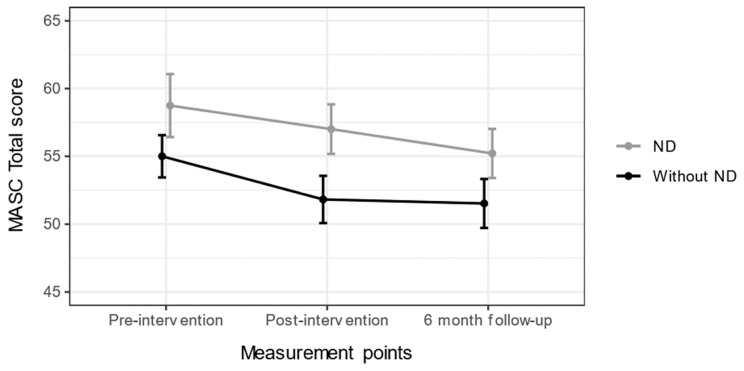
Mean changes (as T-scores) with standard error of means for MASC Total score, based on neurodevelopmental disorder (ND) status for all measurement points.

Changes in MASC Total score based on ND status are shown in Figure 2. The means of the MASC Total score did not show elevated anxiety. No significant interaction effect was detected between ND status and changes in MASC Total score *F*(2, 138) = 0.213, *p* = 0.808. The main effect of ND status was not significant between measurement points, *F*(1, 69) = 3.848, *p* = 0.054. However, the main effect of time was significant between measurement points, *F*(2, 138) = 3.830, *p* = 0.024. Pairwise comparisons did not reach significance.

**Figure 3:**
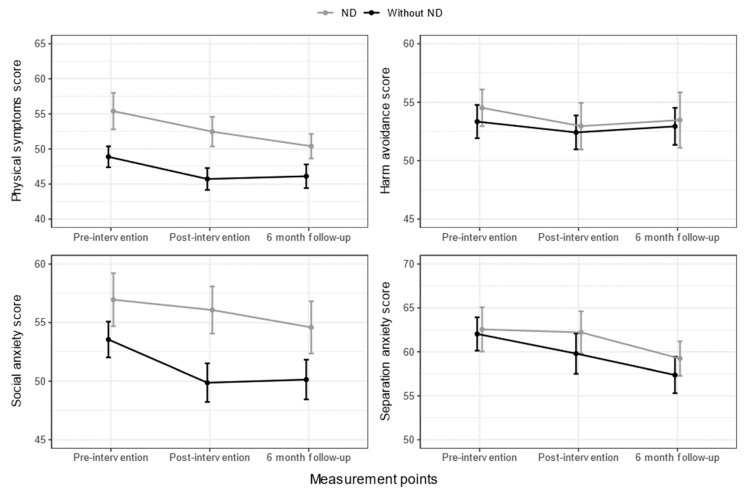
Mean changes (as T-scores) with standard error of means for the MASC subscales, based on neurodevelopmental disorder (ND) status for all measurement points.

As seen in Figure 3, MASC T-scores for children with ND revealed high average to average scores for the *physical symptoms* and *social anxiety* subscales. T-scores for the *separation anxiety* subscale were in the slightly elevated to high average range, regardless of ND status. T-scores for the *harm avoidance* subscale were in the average range for children, regardless of ND status.

Interaction effects were not significant between time and ND on any of the MASC subscales: *physical symptoms F*(2, 138) = 0.492, *p* = 0.612, *harm avoidance F*(2, 138) = 0.040, *p* = 0.961, *social anxiety F*(2, 138) = 0.719, *p* = 0.489, and *separation anxiety F*(2, 138) = 0.241, *p* = 0.786. The main effect for time on *physical symptoms* revealed a significant difference in means at different measurement points *F*(2, 138) = 4.440, *p* = 0.014. The main effect for ND-status also revealed a significant difference in means between children with and without ND *F*(1, 69) = 7.765, *p* < 0.001). The main effect for time on *harm avoidance* was not significant *F*(2, 138) = 0.451, *p* = 0.638, and neither was the main effect for ND status *F*(1, 69) = 0.154, *p* = 0.696. The main effect for time on *social anxiety* revealed significantly different means between measurement points *F*(2, 138) = 3.344, *p* = 0.038. The main effect for ND status also revealed a significant difference in means based on ND status *F*(1, 69) = 4.219, *p* = 0.044). The main effect for time on *separation anxiety* revealed significantly different means between measurement points *F*(2, 138) = 4.229, *p* = 0.016. However, the main effect for ND status did not reveal a significant difference in means between children with and without ND *F*(1, 69) = 0.356, *p* = 0.553).

## 4. Discussion

The present study aimed to investigate changes in anxiety scores during Taste Education intervention for fussy eating, a sensory-based, non-invasive, food-based intervention for children with and without neurodevelopmental disorders (ND). Overall, none of the mean MASC T-scores were elevated as based on MASC T-score guidelines [58]. Participation in the Taste Education study did not raise children’s anxiety, and no significant differences were detected between children’s MASC scores based on ND status. Consistent with our previous findings, no significant change was found in the delayed intervention group between baseline and pre-intervention in relevant outcome measures which indicates that changes in participants’ MASC scores were not explained by time only [45,57].

### 4.1. Changes in Anxiety Scores

The present study’s first aim was to measure changes in MASC scores during the Taste Education intervention. We hypothesized that MASC scores would not increase following the intervention, and although changes were modest, our hypothesis was supported. Our intervention did not explicitly focus on reducing anxiety as such, as our main priority was monitoring changes in mealtime behavior and fussy eating following the intervention [44,45]. Importantly, however, children’s MASC scores were not elevated or increased despite being exposed to the methods used in the Taste Education intervention, i.e., centering on food and food-related stimuli. The current study showed that although the children’s mean MASC Total scores were not elevated, they reduced significantly from high average at pre-intervention to average at post-intervention. Additionally, a six-month follow-up assessment showed a plateau in scores. The changes were significant for the main measurement of anxiety (MASC Total score) and *physical symptoms* of anxiety, *social anxiety*, and *separation anxiety*. The reduction in scores might point to positive shifts in uneasiness around food which might correspond with positive changes in fussy eating, as seen in some studies [44,46].

The lack of elevated MASC scores was surprising as research points to children with fussy eating often presenting with elevated anxiety symptoms [15,46]. However, the children in our study had high socio-economic status (SES) backgrounds and might have had better access to mental health services than those in lower SES brackets. As a substantial proportion of parents in Iceland have to pay out of pocket for their children’s mental health services, bar acute psychiatric episodes [64,65], it is possible that children with higher anxiety levels had not received any psychiatric help, and thus were not as well equipped to participate as the children in our study. None of our participating children took medication for anxiety, which, therefore, did not explain why MASC scores were not elevated.

Changes on the *harm avoidance* subscale were not significant, although the lack of significant differences could reflect the poor internal consistency for the *harm avoidance* subscale, which has also been reported in other studies [59]. Therefore, it might be misleading to interpret these results. Interestingly, the highest mean scores were seen for the *separation anxiety* subscale, with a significant reduction between measurement points (slightly elevated to average). The reason for the significant reduction is not immediately apparent but might be explained by the parent–child interactions [8,46,66] in which the children had to gradually increase their independence during the kitchen session with other children, but always with a taste educator on hand.

The reduction in the *social anxiety* subscale was significant between measurement points. However, means reduced from high average scores at pre-intervention and average scores at six-month follow-up; so, despite the significant difference, the clinical relevance is uncertain as none of the mean scores were elevated. Our study centered on a group format, so children were required to interact with other children and adults, including parents and taste educators, performing several tasks in the group setup, albeit always in a supportive and understanding environment. Therefore, despite children not having elevated social anxiety, the intervention format, importantly, did not raise children’s anxiety levels.

### 4.2. Changes in Anxiety Scores Based on Neurodevelopmental Disorder Status

The study’s second aim was to compare changes in anxiety scores in children with and without ND. We hypothesized that anxiety scores in children with ND would reduce less than in children without ND. This hypothesis was not supported. Although there were higher scores for children with ND, and significant differences in mean MASC Total T-scores and subscales such as *social anxiety* and *separation anxiety* between children with and without ND, the interaction effects between ND status and changes in scores over time were not significant. This suggests that children with ND did not fare significantly worse in terms of MASC scores after the Taste Education intervention. The non-difference is incongruent with previous research on the prevalence of anxiety among children with ND [37,40,42]. However, children with ASD might experience anxiety symptoms differently than TD children, i.e., considering their sensory issues, and they may not be able to express anxiety symptoms as vocally, relying more on ticks or stimming (repeating noises or movements for coping in overwhelming situations) instead [12,19,47,67]. Although we did not notice frequent stimming in children with ASD, we did allow those who were feeling overwhelmed to withdraw to a quiet area; however, we kept the kitchen doors open so that the smells and muffled sounds did carry for gentle exposure. Of the two children that opted for quiet time, neither chose to stay for longer than 15 min. Further, the MASC has not been standardized for children with ND [68]. The unstandardized measurement for children with ND might explain the lack of significant differences between the groups, or the low statistical power as the sample size was small.

There was a significant difference between children with and without ND on the *physical symptoms* subscale. However, although none of the children had elevated T-scores, the differences between children based on ND status are not specific to our study sample, as measures of physical anxiety symptoms are generally higher in children with ND [41].

T-scores for *separation anxiety* were not significantly different between children based on ND status. Although they were not elevated, they were in the slightly elevated to high average range. It is not immediately clear why the scores were raised at pre-intervention. The children may have been apprehensive about the intervention when it was initially explained by the parents; keep in mind that the Cronbach’s alpha for the *separation anxiety* subscale is low on the Icelandic scale so not possible to interpret fully. We did not find any studies supporting increased separation anxiety in children with ND in a similar situation.

Anxiety is considered an underlying and often mediating factor in fussy eating [16,46,56,69]. Although the mean MASC Total score did not reveal elevated anxiety levels, during the intervention, signs of fear were markedly exhibited in some of the participating children, who were visibly upset at the beginning of our kitchen sessions when they noticed fruit, vegetables, seeds, and nuts on the workstations. They were even more suspicious of what they were not seeing since some of the children had experienced being served prepared meals with hidden ingredients, such as pureed vegetables. This method is common among parents and other caregivers when “sneaking” nutritious foods into their children’s diets, such as squash in muffins or vegetables in fruit smoothies, but it does little to reduce children’s unease towards food as they are not gently and repeatedly exposed to the feared stimuli. Further, children might experience disgust from the visual perception of food [70], especially from the sensory aspects of soft or fibrous textures, rather than the taste itself [71,72], which underscores the importance of educating parents on their children’s responses and reactions to feared foods.

The results of our previous papers [44,45] combined with our current results indicate that the methods used in the intervention are suitable for children with ND, and even though we did not directly address anxiety symptoms, our repeated exposure methods are similar to those used in other treatments for anxiety disorders such as phobias, specifically for those with ND [33,73,74,75].

### 4.3. Strength and Limitations

The current study had many strengths. It was unique in that it included children with and without ND and measured changes in anxiety scores over time during a food-based intervention. The study included simple methods which are easy to implement, and the drop-out rate was low, suggesting a good acceptance of the study among participants. The study was inclusive, with participants presenting various conditions and diagnoses. Measurements at six-month follow-up showed that, although modest, changes in anxiety scores were generally maintained, suggesting positive effects in the long term. There were also no significant differences between the immediate and delayed interventions, suggesting that the changes were not based on time alone.

Some study limitations should also be mentioned. Although the study was inclusive, it only included children with higher-functioning ASD, so the results may not be replicable for all children with ASD. A large proportion of parents had completed higher education, so the results may not be replicable for populations with lower SES status, specifically since those in lower SES situations may not be able to seek professional assistance for their child’s mental health due to financial restraints, and they might, therefore, have higher MASC scores than our participants. The questions on the MASC are not tailored towards anxiety over food per se, limits to a child’s diet, or food neophobia. For further studies it might be beneficial to use measurements designed more towards food anxiety, particularly measuring fear responses regarding eating and handling food, as well as specific questions on food neophobia. The MASC is also not standardized for children with ASD, and since the measurement is self-reported, additional objective observations or even functional analyses would be helpful. Although changes in MASC Total scores and some of the subscales were statistically significant, mean MASC Total scores and some of the subscales were not elevated which limits the generalizability of the study for children with anxiety and fussy eating. Lastly, the sample size was small and homogenous, and effect sizes were small.

## 5. Conclusions

Participation in the Taste Education study did not raise children’s anxiety levels but did lower anxiety scores between measurement points, providing guarded optimism for positive shifts towards unease around previously disliked foods following a food-based intervention. The results were similar regardless of ND status. These findings support the need for investigating anxiety in children with fussy eating and how best to serve children with and without ND. Further studies with larger and more divergent samples are necessary.

## Figures and Tables

**Table 1:** Characteristics of children who completed all measurement points on the Multidimensional Anxiety Scale for Children (MASC) and their parents, comparing participants based on neurodevelopmental disorder (ND) status.

	Total Sample (*n* = 71)	Without ND (*n* = 40)	ND (ADHD, ASD, or Both) (*n* = 31)
**Child**			
Female, *n* (%)	28 (39.4)	20 (50.0)	8 (25.8)
Male, *n* (%)	43 (60.6)	20 (50.0)	23 (74.2)
Mean age in years (SD)	9.2 (1.58)	8.9 (1.63)	9.4 (1.48)
**Diagnosis**, *n* (%)			
ADHD, primarily	11 (15.5)	-	11 (35.5)
ASD, primarily	5 (7.0)	-	5 (16.1)
Anxiety	8 (11.3)	2 (5.0)	6 (19.3)
Other	7 (9.8)	4 (10.0)	3 (9.7)
**Parent**			
Mother	65 (91.5)	37 (92.5)	28 (90.3)
Father	6 (8.5)	3 (7.5)	3 (9.7)
**Education level**, *n* (%)			
No higher education	4 (5.6)	3 (7.5)	1 (3.2)
Vocational education	11 (15.5)	5 (12.5)	6 (19.4)
University level	56 (78.9)	29 (72.5)	27 (87.1)
**Occupational status**, *n* (%)			
Full-time occupation	54 (76.1)	33 (82.5)	21 (67.7)
Part-time occupation or student	17 (23.9)	10 (25.0)	7 (22.6)
**Single-parent household**, *n* (%)	10 (14.1)	3 (7.5)	7 (22.6)
**Children in the household**, *n* (%)			
1	8 (11.3)	4 (10.0)	4 (12.9)
2	30 (42.3)	17 (42.5)	13 (41.9)
3 or more children	33 (46.5)	19 (47.5)	14 (45.2)

Abbreviations, *n* sample size, SD standard deviation, ND neurodevelopmental disorder, ASD Autism Spectrum Disorder, ADHD Attention Deficit/Hyperactivity Disorder.

**Table 2:** Repeated measures analysis of variance (ANOVA) showing changes in means based on MASC at all measurement points: pre-intervention, post-intervention, and six-month follow-up. Means are shown as T-scores.

MASC Total *n* = 71	Pre-Intervention *M* (SD)	Post-Intervention *M* (SD)	6 Month Follow-Up *M* (SD)	*F*	*p*	Cohen’s d
MASC Total	56.6 (11.38)	54.1 (10.89)	53.1 (10.93)	3.987	0.021	0.32
Physical symptoms	51.7 (12.25)	48.7 (11.17)	47.9 (10.42)	4.323	0.015	0.33
Harm avoidance	53.8 (8.84)	52.6 (9.99)	53.2 (11.44)	0.434	0.649	0.06
Social anxiety	55.0 (11.10)	52.8 (11.12)	52.1 (11.60)	3.683	0.028	0.26
Separation anxiety	62.3 (12.87)	60.8 (13.97)	58.2 (12.11)	4.488	0.013	0.33

Abbreviations, *n* sample size, M mean, SD standard deviation, F F-ratio, *p p*-value, d effect size, MASC Multidimensional Anxiety Scale for Children. Values in bold font indicate significant differences. T-score guidelines: <40 = low, 40–54 = average, 55–59 = high average, 60–64 = slightly elevated, 65–69 = elevated, and ≥70 very elevated [58].

## Data Availability

The data presented in this study are available on request from the corresponding author.
